# Supplementary material for: The dopamine transporter antiports potassium to increase the uptake of dopamine
Source: Nat Commun. 2022 May 4;13:2446. doi: 10.1038/s41467-022-30154-5 (PMC9068915; doi:10.1038/s41467-022-30154-5)
Supplement: Supplementary file 1 — Supplementary Information [file 41467_2022_30154_MOESM1_ESM.pdf]

SUPPLEMENTARY INFORMATION for:

The dopamine transporter antiports potassium to increase the uptake of dopamine

Solveig G. Schmidt[1], Mette Galsgaard Malle[2,3], Anne Kathrine Nielsen[1,4], Søren S.-R. Bohr[2,3], Ciara F. Pugh[1], Jeppe C. Nielsen[1], Ida H. Poulsen[1], Kasper D. Rand[4], Nikos S. Hatzakis[2,3], Claus J. Loland[1,*]

[1]Laboratory for Membrane Protein Dynamics, Department of Neuroscience, Faculty of Health and Medical Sciences, University of Copenhagen, Copenhagen, Denmark.

[2]Nano-Science Center, Department of Chemistry, Faculty of Science, University of Copenhagen, Copenhagen, Denmark

[3]Novo Nordisk Foundation Center for Protein Research, Faculty of Health and Medical Sciences, University of Copenhagen, Copenhagen, Denmark

[4]Protein Analysis Group, Department of Pharmacy, Faculty of Health and Medical Sciences, University of Copenhagen, Copenhagen, Denmark.

These authors contributed equally: Mette Galsgaard Malle, Anne Kathrine Nielsen

*Correspondence:
Claus J. Loland, Department of Neuroscience, University of Copenhagen, Denmark,
cllo@sund.ku.dk

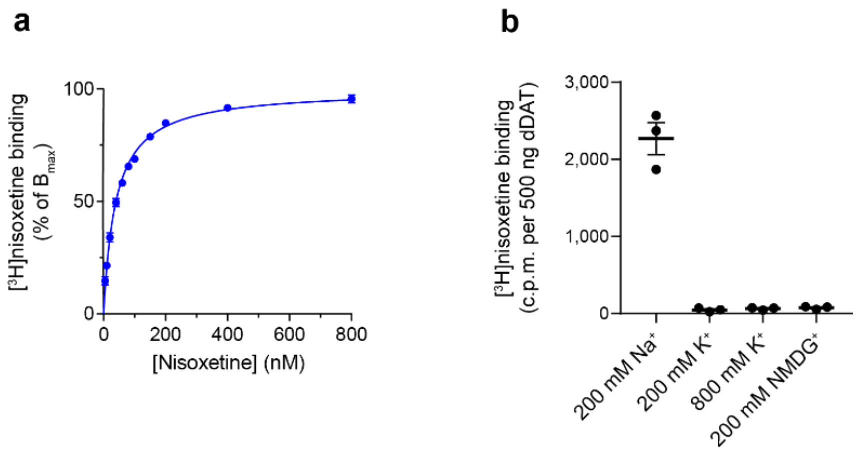

Supplementary Figure 1. [^{3}H]nisoxetine binding to detergent solubilized dDAT. a Saturation binding of [^{3}H]nisoxetine to dDAT in a buffer containing 200 mM NaCl showed a K_d of 40 ± 2 nM (means ± SEM). Data are shown as mean ± SEM (error bars) and fitted to a one-site specific binding curve using GraphPad Prism 7.0. n = 4 independent experiments performed in duplicates. **b** Binding of 120 nM [^{3}H]nisoxetine to dDAT in indicated salt conditions. Specific binding was observed in Na^+, whilst all other salt conditions did not promote [^{3}H]nisoxetine binding above background (1 mM nortriptyline). The ionic concentration was kept constant by substitution of Na^+ and K^+ with $NMDG^+$. Data points represent mean ± SEM (error bars), n = 3 independent experiments performed in duplicates. Data are provided as a Source Data file.

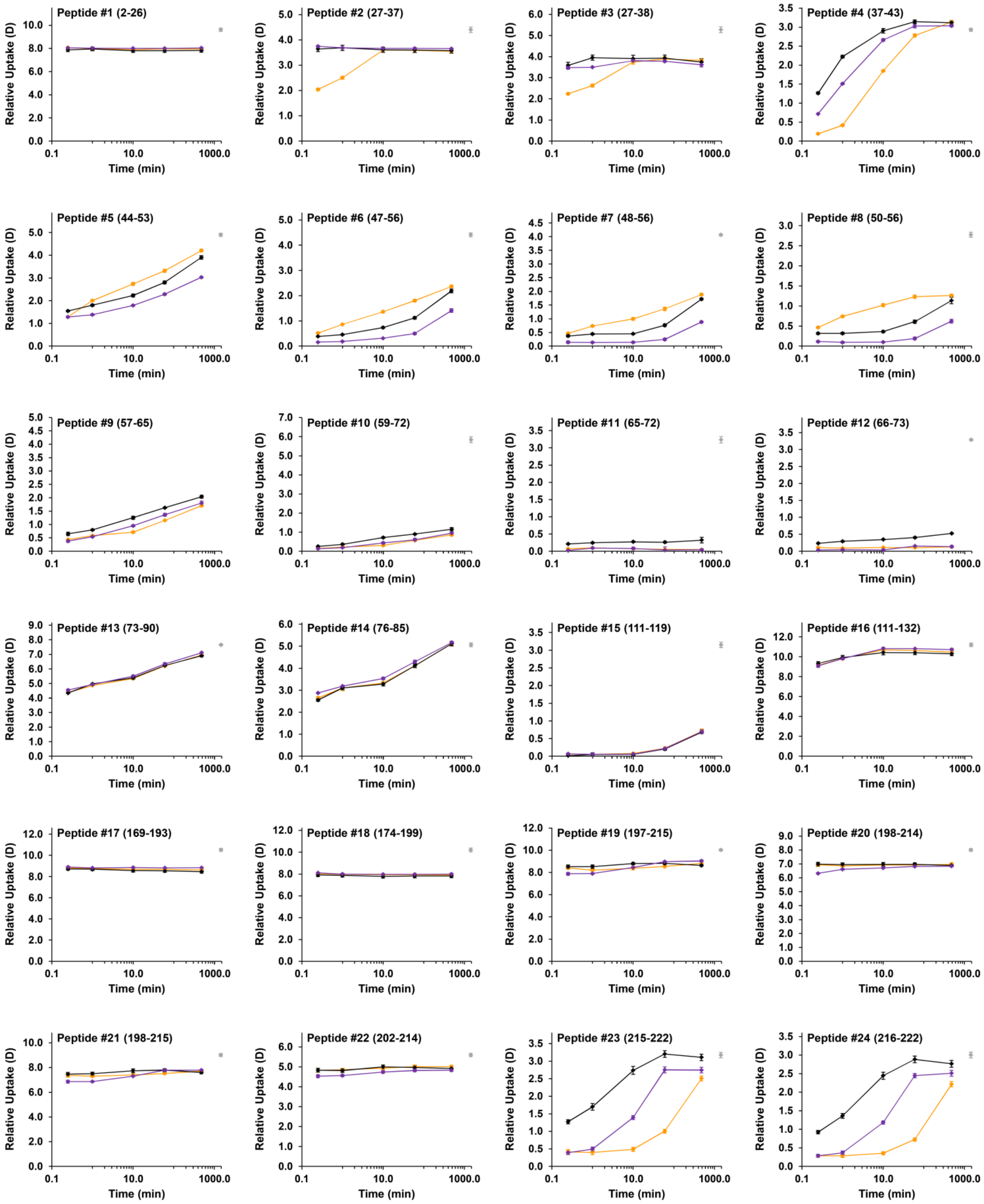
Peptide #1 (2-26)
Peptide #2 (27-37)
Peptide #3 (27-38)
Peptide #4 (37-43)
Peptide #5 (44-53)
Peptide #6 (47-56)
Peptide #7 (48-56)
Peptide #8 (50-56)
Peptide #9 (57-65)
Peptide #10 (59-72)
Peptide #11 (65-72)
Peptide #12 (66-73)
Peptide #13 (73-90)
Peptide #14 (76-85)
Peptide #15 (111-119)
Peptide #16 (111-132)
Peptide #17 (169-193)
Peptide #18 (174-199)
Peptide #19 (197-215)
Peptide #20 (198-214)
Peptide #21 (198-215)
Peptide #22 (202-214)
Peptide #23 (215-222)
Peptide #24 (216-222)
Relative Uptake (D)
Time (min)

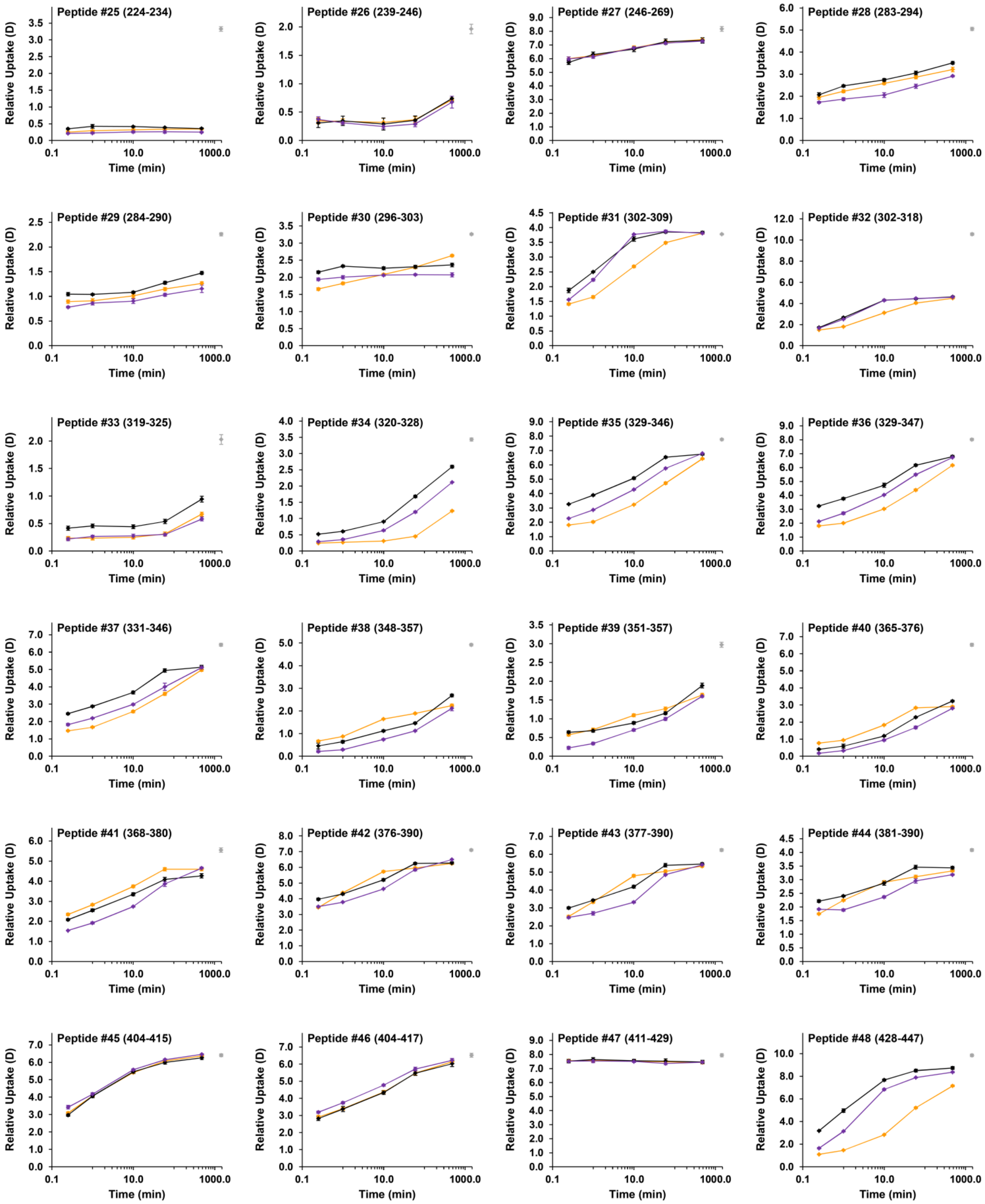
Peptide #25 (224-234)
Peptide #26 (239-246)
Peptide #27 (246-269)
Peptide #28 (283-294)
Peptide #29 (284-290)
Peptide #30 (296-303)
Peptide #31 (302-309)
Peptide #32 (302-318)
Peptide #33 (319-325)
Peptide #34 (320-328)
Peptide #35 (329-346)
Peptide #36 (329-347)
Peptide #37 (331-346)
Peptide #38 (348-357)
Peptide #39 (351-357)
Peptide #40 (365-376)
Peptide #41 (368-380)
Peptide #42 (376-390)
Peptide #43 (377-390)
Peptide #44 (381-390)
Peptide #45 (404-415)
Peptide #46 (404-417)
Peptide #47 (411-429)
Peptide #48 (428-447)
Relative Uptake (D)
Time (min)

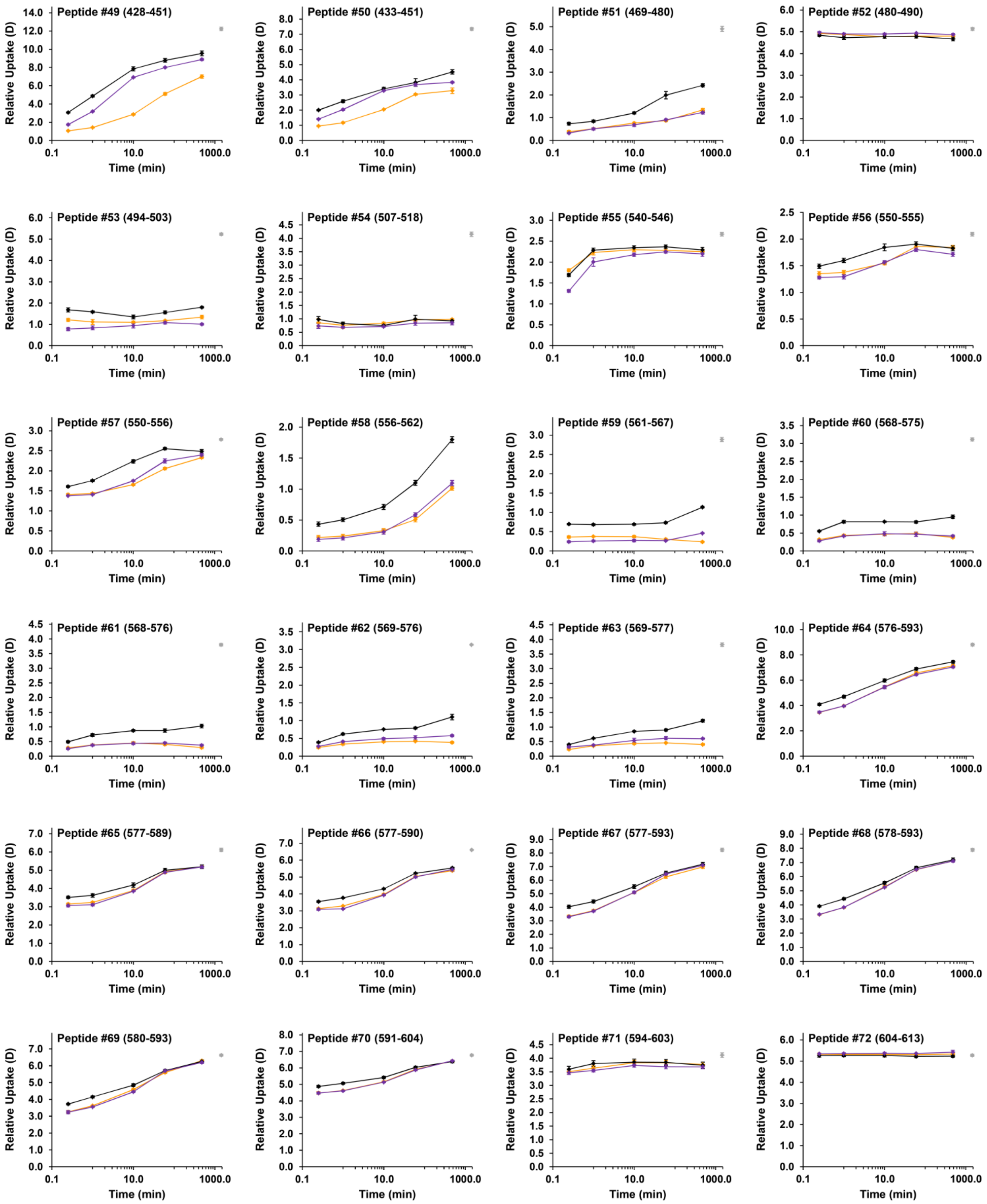
Peptide #49 (428-451)
Peptide #50 (433-451)
Peptide #51 (469-480)
Peptide #52 (480-490)
Peptide #53 (494-503)
Peptide #54 (507-518)
Peptide #55 (540-546)
Peptide #56 (550-555)
Peptide #57 (550-556)
Peptide #58 (556-562)
Peptide #59 (561-567)
Peptide #60 (568-575)
Peptide #61 (568-576)
Peptide #62 (569-576)
Peptide #63 (569-577)
Peptide #64 (576-593)
Peptide #65 (577-589)
Peptide #66 (577-590)
Peptide #67 (577-593)
Peptide #68 (578-593)
Peptide #69 (580-593)
Peptide #70 (591-604)
Peptide #71 (594-603)
Peptide #72 (604-613)
Relative Uptake (D)
Time (min)

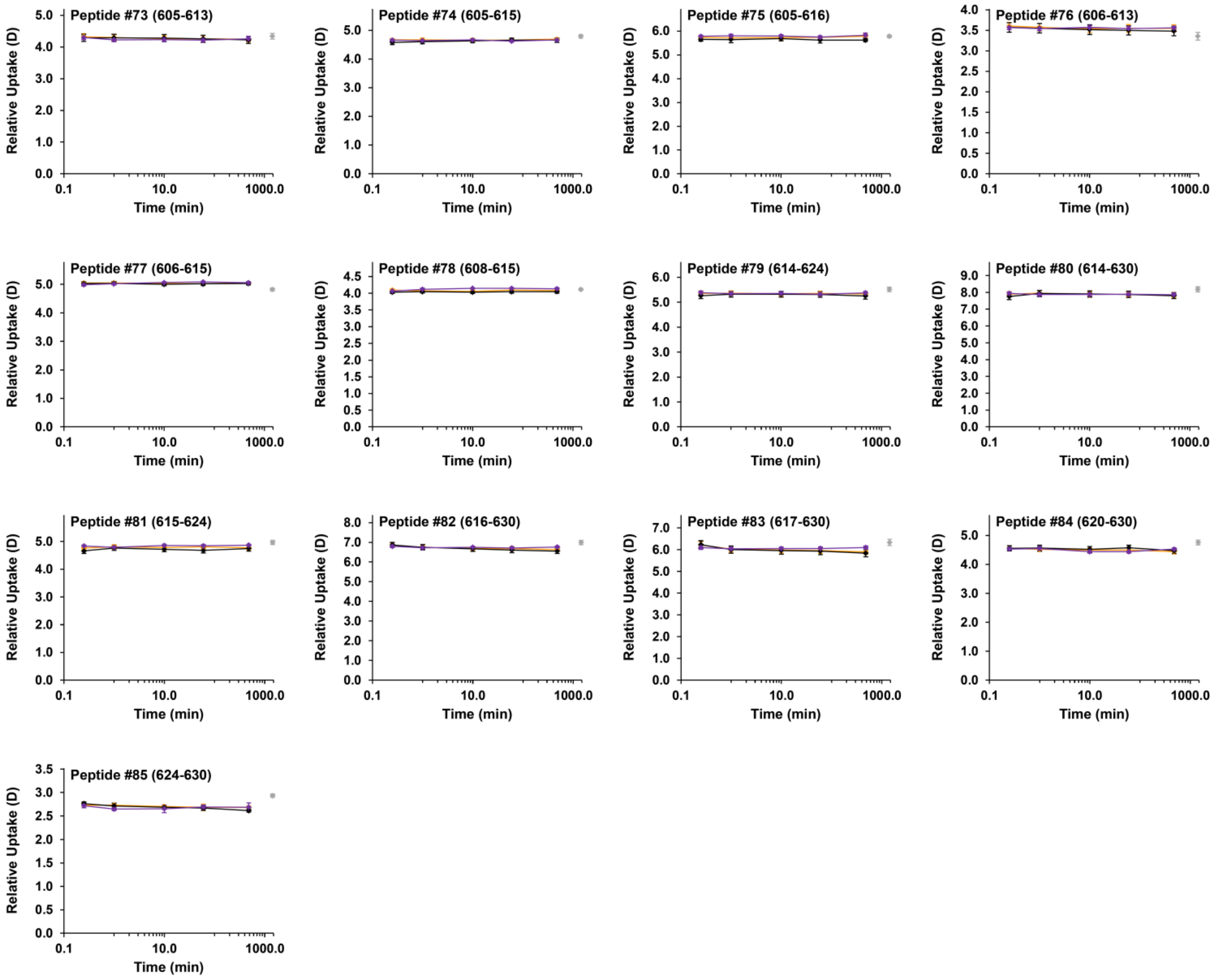

Supplementary Figure 2. Deuterium uptake plots for dDAT peptides. Deuterium uptake plots for representative peptides of dDAT showing the relative deuterium uptake as a function of labeling time (0.25 – 480 min) for the K$^+$ (purple), Cs$^+$ (black), and Na$^+$-bound (orange) states. Values represent means of three (n = 3 for Cs$^+$ and K$^+$) or six (n = 6 for Na$^+$) independent measurements with SEM values plotted as error bars but these are in most instances too small to be visible. Maximum-labeled control samples are shown as grey circles at 1440 min. Data for the Cs$^+$ and Na$^+$-bound states as well as for the maximum labeled control samples were published previously[1], but acquired together with the data for the K$^+$ state and shown here for comparison. Source data are provided as a Source Data file.

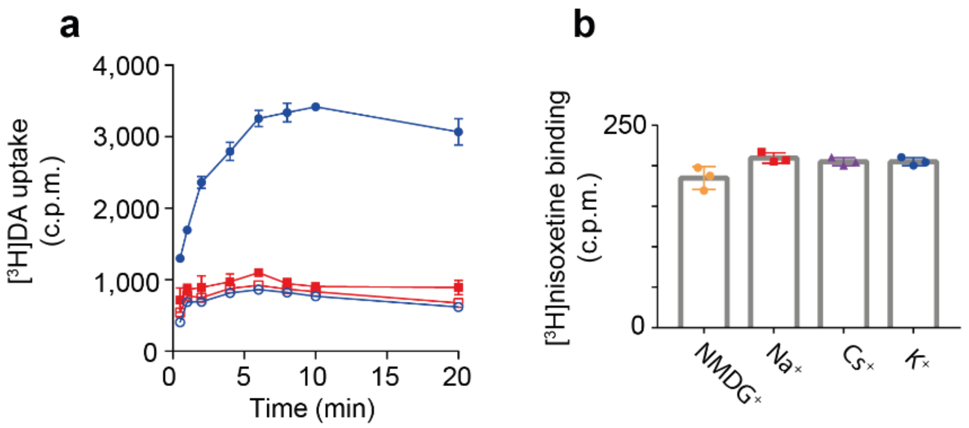

Supplementary Figure 3. [^{3}H]dopamine uptake in proteoliposomes and [^{3}H]nisoxetine binding to dDAT from solubilized proteoliposomes. a Uncorrected data of dDAT [^{3}H]dopamine uptake into PLs in uptake buffer containing an intra-vesicular buffer with either 200 mM K^+ (filled blue circles) or 200 mM Na^+ (red filled squares), or with 100 µM nortriptyline added to the uptake buffer (K^+: open blue circles, Na^+: open red squares). **b** Binding of [^{3}H]nisoxetine to dDAT from re-solubilized PLs was measured in saturating conditions (approximately 20x K_d). Data in (a) and (b) are shown as mean ± S.D. (error bars), from one representative experiment performed in triplicates. Data are provided as a Source Data file.

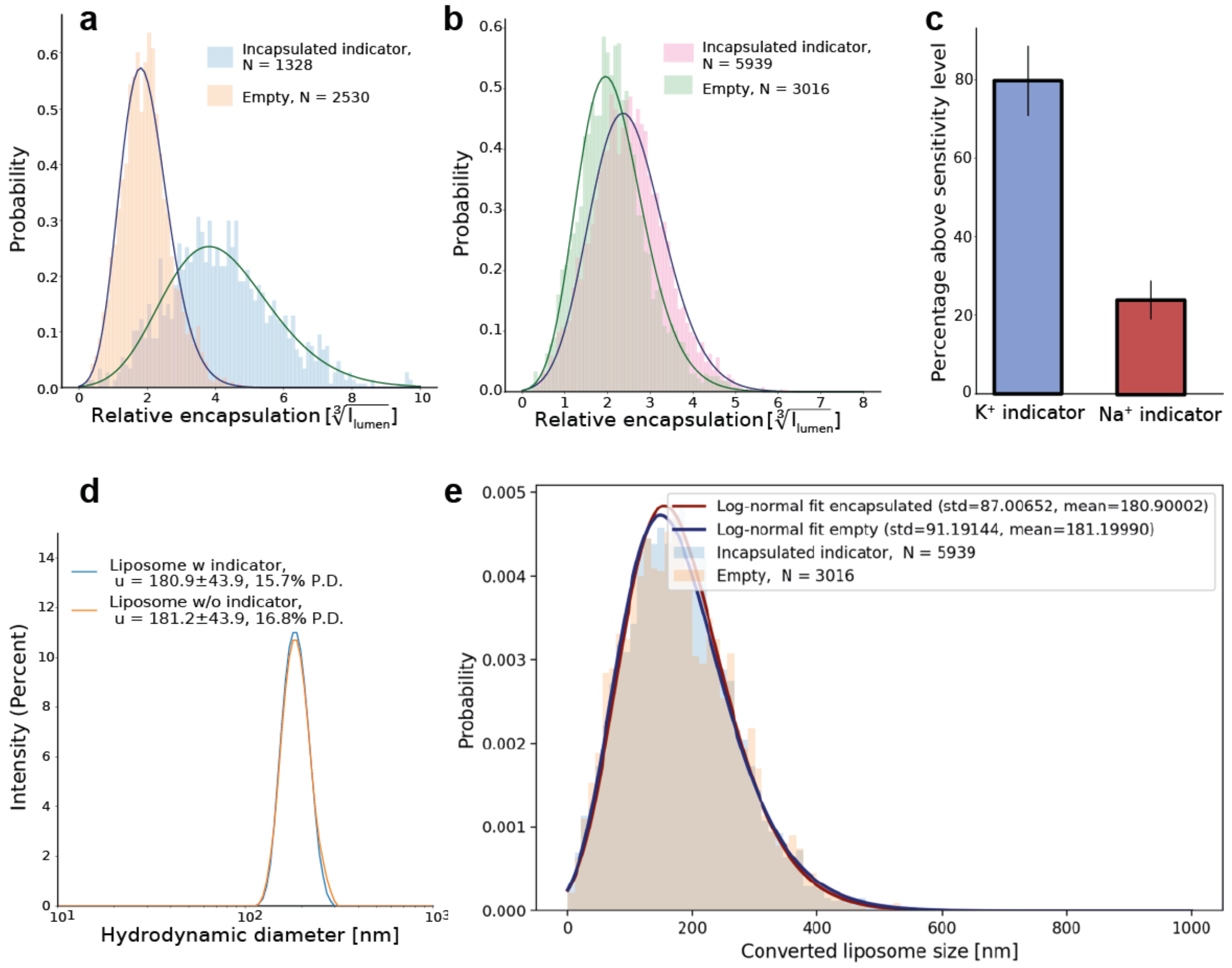

Supplementary Figure 4. Encapsulation of the fluorescent indicators and assessment of liposome size. Quantitative calibration of the encapsulation efficiency of both the fluorescent K^+ indicator and the fluorescent Na^+ indicator, to quantify the sensitivity of the system, and thereby the upper percentage of liposomes with sufficient indicator to enable a quantitative measurable signal in the real time TIRF monitoring. **a** Empty (indicator-free) PLs and PLs with 10 mM K^+ indicator (n = 2530 and 1328 liposomes, respectively) imaged with identical settings and co-localized for quantifications of encapsulated dye signal. By comparing the background crosstalk signal and the indicator signal we found 79.8 ± 8.9% (mean ± SEM) of the PLs to have measurable signal sensitivity and encapsulation,. **b** Empty PLs and PLs with 10 mM Na^+ indicator (n = 3016 and 5939 liposomes, respectively) imaged with identical settings. Comparison showed 24.2 ± 4.9% of the PLs had measurable signal sensitivity and encapsulation,. **c** Integration of the crosstalk signal from the membrane dye and the indicator signal for both conditions (10 mM K^+ indicator with 100 mM K^+, 100 mM $NMDG^+$ intra-vesicular concentration, and 10 mM Na^+ indicator with 200 mM Na^+ intra-vesicular concentration) as depicted in panels a and b gives a quantification of the percentage of PLs with sensitive settings allowing the direct observation of respectively uptake of Na^+ and outflow of K^+.

Error bars reflect the standard deviation assumed that the data follows a Poisson distribution. The K^+ indicator assay allowed an upper quantification percentage of 79.8 ± 8.9% of the individual PLs, and the Na^+-indicator assay allowed an upper quantification percentage of 24.2 ± 4.9% of the individual PLs. The upper quantification percentage is the maximum percentage of liposomes with sufficient indicator to enable a quantitative measurable signal in the TIRFm set-up. **d** DLS measurement of PLs reconstituted with fluorescent indicator in the lumen (blue) and without (orange) showing that the indicator does not influence the size (u) or polydispersity (P.D.) of the liposomes. **e** Integrated membrane signal from 5939 liposomes with encapsulated indicator (red) and 3016 without encapsulated indicator (blue). The square root of the membrane integrated signal is proportional to the liposome size, and shows a lognormal distribution of sizes as expected. Using the mean liposome size evaluated by DLS, the membrane intensity can be converted to liposome sizes in nm. We find no significant difference in liposome sizes with and without encapsulated indicators. Data are provided as a Source Data file.

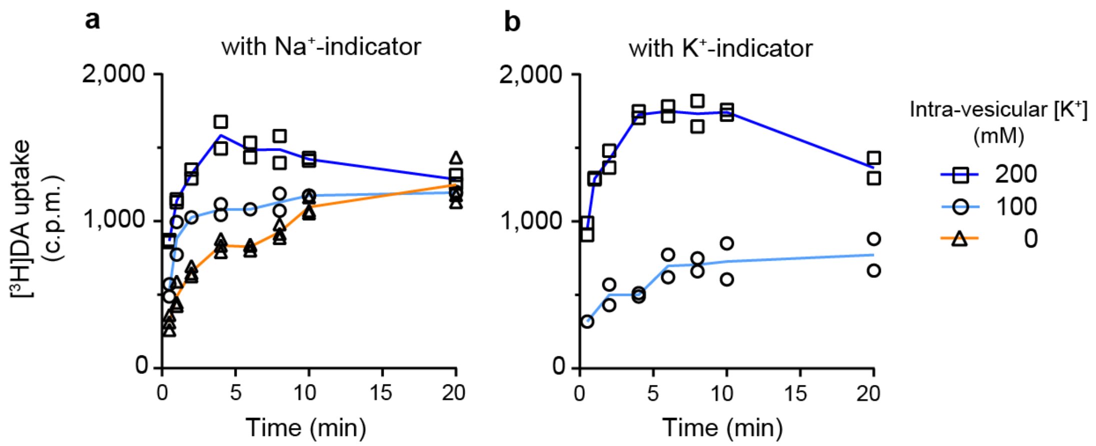

Supplementary Figure 5. [^{3}H]dopamine uptake in proteoliposomes reconstituted with membrane dye, biotinylated PEG-DOPE and encapsulating ion indicator. PLs with both Na^+ indicator (**a**) and K^+ indicator (**b**) showed Na^+ dependent [^{3}H]dopamine uptake with varying [K^+] in the lumen. Data are shown from one representative experiment performed in duplicates. Data are provided as a Source Data file.

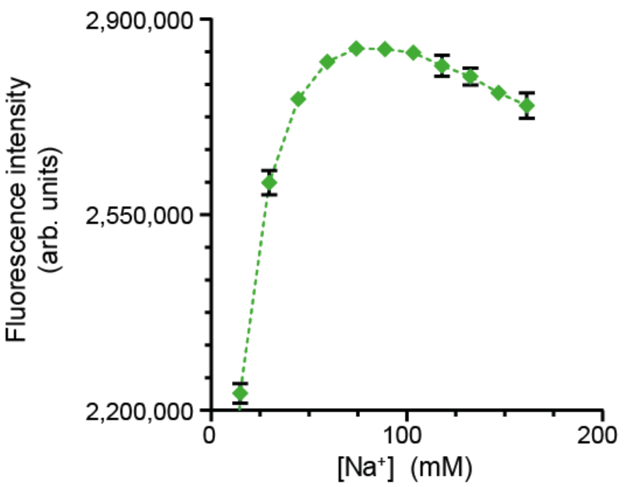

Supplementary Figure 6. Sensitivity of the fluorescent Na^+ indicator. Na^+ indicator (10 mM) was excited at 503 nm and fluorescence was measured at maximum emission (529 nm) in increasing [NaCl] (0-160 mM) in buffer containing 20 mM HEPES (pH 7.5) and 1 mM L-ascorbic acid. The maximal fluorescence intensity was reached at 75 mM NaCl. The fluorescence signal obtained in 160 mM matched the signal intensity at 45 mM. Data are shown as mean ± S.D. (error bars), n = 1 experiment performed in triplicates. Data are provided as a Source Data file.

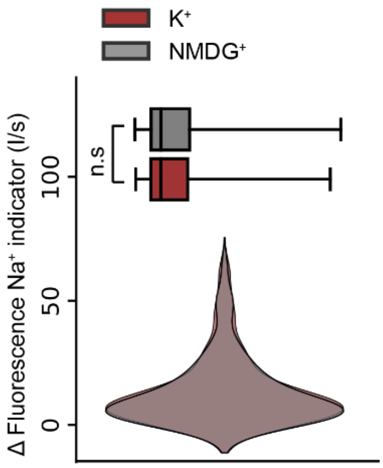

Supplementary Figure 7. Translocation kinetics of Na$^+$ in absence of dopamine. (**Bottom panel**) Overlay of violin plots displaying Na$^+$ uptake rates obtained in the absence of dopamine in vesicles containing intra-vesicular K$^+$ or NMDG$^+$, respectively. (**Top panel**) Box plots showings the statistical dispersion of the fluorescence signal increase rates from all single vesicle data in the violin plots. The box plots shown the minimum (min), maximum (max), median (med), quartile (25%) Q1 (q1) and quartile (75%) Q3 (q3). The upper whiskers equal to upper bound and maxima and lower whiskers equal to lower bound and minima to show the full distribution. The value for min, max, med, q1 and q3 in the box plots are reported in Supplementary Table 7. Comparing the effect of Na$^+$ uptake revealed no significant rate difference in the absence of dopamine validated by a two-sided KS-test ($P = 0.946$). This suggest that the continuous translocation of Na$^+$ across vesicle membranes is independent of intra-vesicular K$^+$. N = 83 liposomes containing K$^+$ and 110 liposomes containing NMDG$^+$ examined in 12 individual experiments each. Data are provided as a Source Data file.

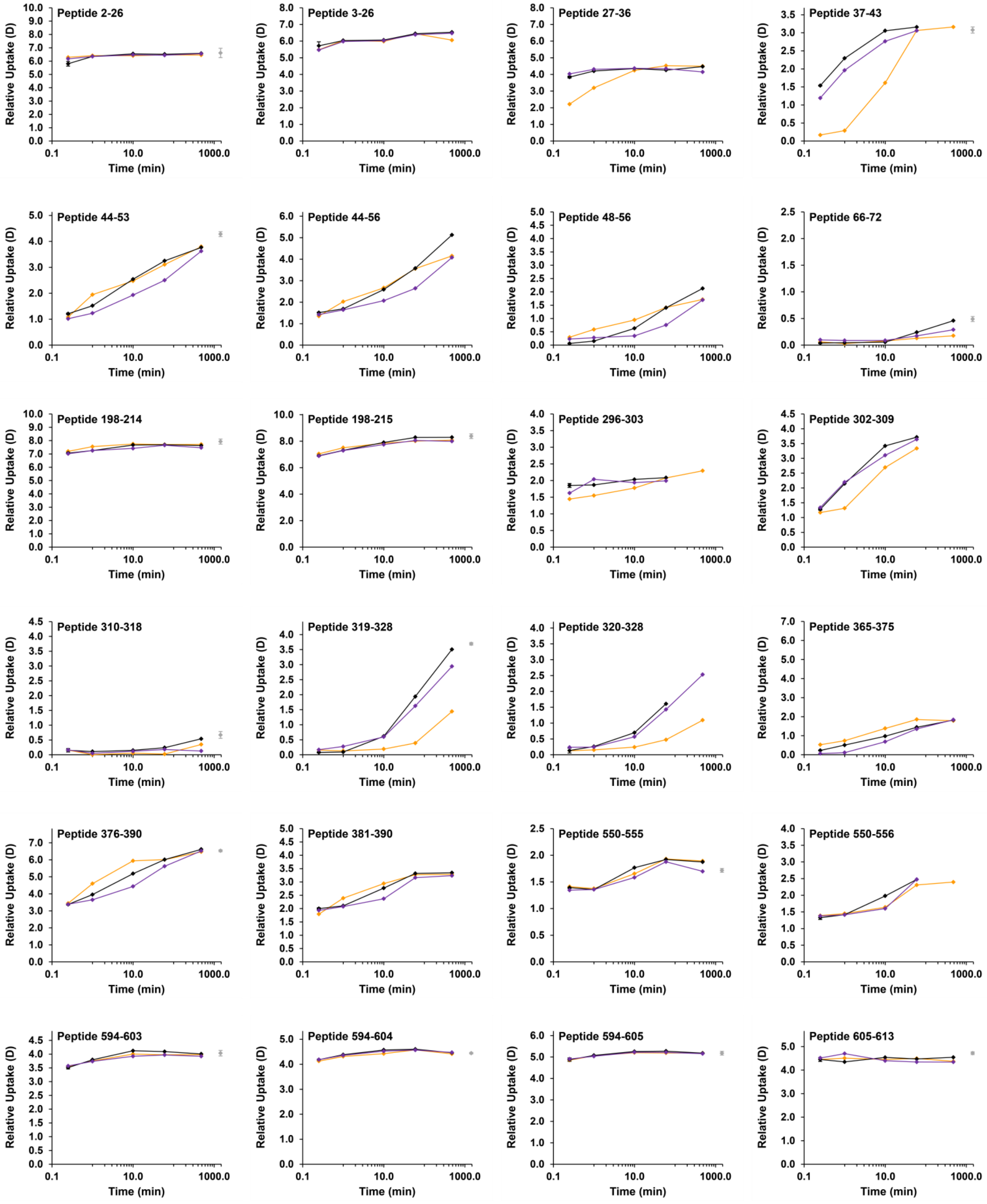
Peptide 2-26
Peptide 3-26
Peptide 27-36
Peptide 37-43
Peptide 44-53
Peptide 44-56
Peptide 48-56
Peptide 66-72
Peptide 198-214
Peptide 198-215
Peptide 296-303
Peptide 302-309
Peptide 310-318
Peptide 319-328
Peptide 320-328
Peptide 365-375
Peptide 376-390
Peptide 381-390
Peptide 550-555
Peptide 550-556
Peptide 594-603
Peptide 594-604
Peptide 594-605
Peptide 605-613
Relative Uptake (D)
Time (min)

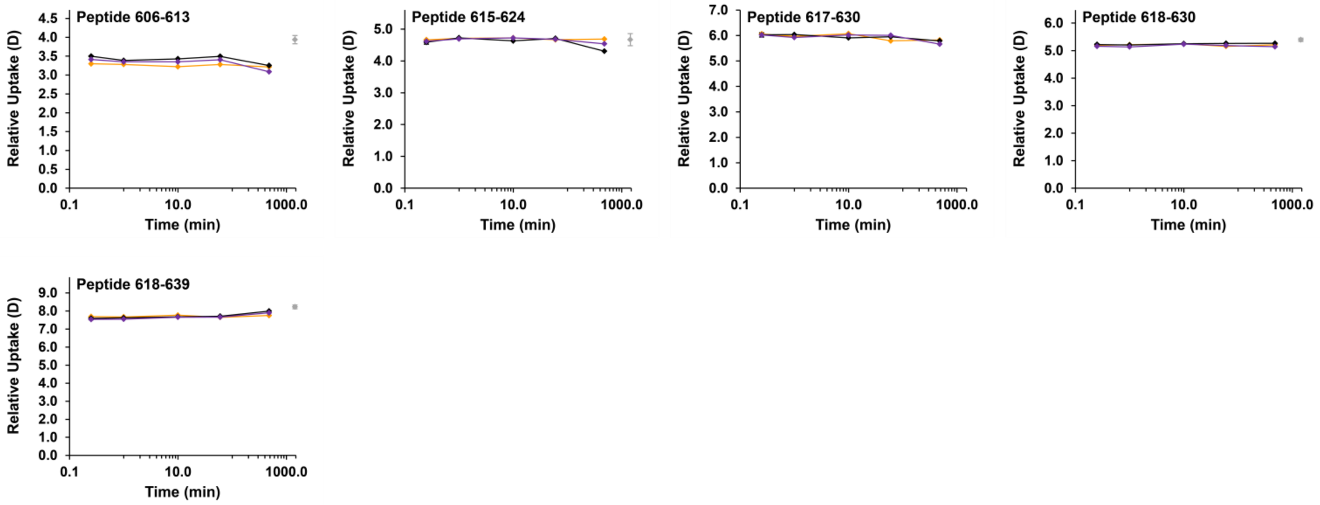

Supplementary Figure 8. Deuterium uptake plots for dDAT peptides in preliminary HDX-MS study. Deuterium uptake plots for representative peptides of dDAT showing the relative deuterium uptake as a function of labeling time (0.25 – 480 min) for the K^+ (purple), Cs^+ (black), and Na^+-bound (orange) states. In the preliminary HDX-MS study, time points were done in singlicate (n = 1) for all states except for the 0.25 min time point of the Cs^+ state, which was done in triplicate (n = 3). Maximum-labeled control samples are shown as grey circles at 1440 min (n = 4). Values are plotted as means with SEM values plotted as error bars for time points where n > 1. Source data are provided as a Source Data file.

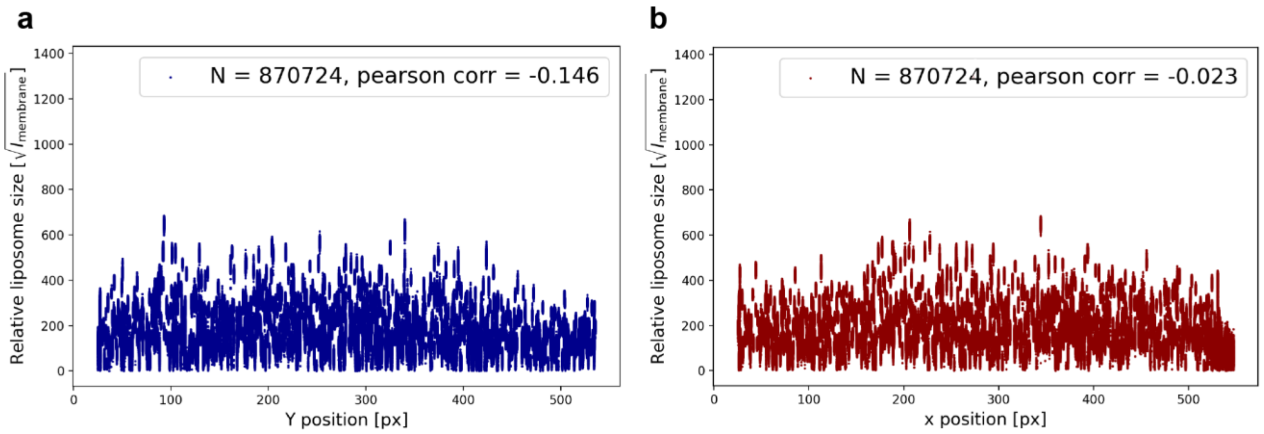

Supplementary Figure 9. Background corrected liposome-membrane intensity assessed for bias in position in field of view. Background corrected liposome-membrane intensity for the entire y positions (**a**) and x positions (**b**) over the field of view from the TIRF microscope. Using the local background correction liposome intensity and evaluation is unaffected by the position in the field of view. n = 870724 liposomes from 6 individual experiments. Data are provided as Source Data file

Supplementary Table 1. Effect of K^+ on Na^+-dependent [^{3}H]nisoxetine binding to dDAT

$[K^+]$ (mM)	B_{max} (normalized)	EC_{50} (mM)	Hill Slope	n
0	97.6 ± 4.1	60 [53;67]	1.96 ± 0.42	3
20	99.3 ± 3.4	68 [62;75]	1.72 ± 0.27	3
50	99.3 ± 4.6	106 [96;118]	1.50 ± 0.24	3
100	94.8 ± 7.4	122 [103;145]	1.54 ± 0.40	3
200	96.8 ± 5.0	170 [154;188]	1.98 ± 0.36	3
400	97.5 ± 3.3	210 [198;222]	2.52 ± 0.34	3
600	99.4 ± 3.2	319 [303;335]	2.39 ± 0.19	3
800	100.0 ± 4.0	436 [416;458]	2.39 ± 0.19	3

The B_{max}, EC_{50} and Hill slopes determined by binding of 120 nM [^{3}H]nisoxetine to dDAT WT in increasing Na^+ in the presence of the indicated K^+ concentrations fitted to a sigmoidal dose-response (variable slope) equation. The ionic strength was maintained by substitution of Na^+ and K^+ with $NMDG^+$. Binding is shown in Fig. 1a. Data are mean ± SEM or [SEM interval].

Supplementary Table 2. HDX Data summary table

Data Set	K^+ state
HDX reaction details	40 mM Tris, 5% glycerol, 1 mM DDM, 0.2 mM CHS, 14 µM lipids (POPC:POPE:POPG; weight ratio 3:1:1), 200 mM KCl. Percent deuterium: 75.2%, pD_{read} = 8.036, 25°C.
HDX time course	0.25 min, 1 min, 10 min, 60 min, 480 min
HDX control samples	Maximum-labeled control using predigested dDAT.
Back-exchange (mean / IQR)	32.1% / 9.55%
# of Peptides	85
Sequence coverage	75.5% (77.2% of the dDAT sequence)
Average peptide length / Redundancy	12.44 / 2.17
Replicates (biological or technical)	3 (technical)
Repeatability	0.0504 (average standard deviation)
Significant differences in HDX (delta HDX > X D)	95% CI (Cs^+-K^+): 0.24 D<br>95% CI (Na^+-K^+): 0.20 D

Summary table of HDX experimental conditions and data.

Supplementary Table 3. HDX data table.

Peptide #	Start	End	Sequence	Peptide mass (Da)	Retention time (min)	Uptake - K+ (D) 0.25 min	Uptake - K+ (D) 1 min	Uptake - K+ (D) 10 min	Uptake - K+ (D) 60 min	Uptake - K+ (D) 480 min	Uptake error (SD) - K+ (D) 0.25 min	Uptake error (SD) - K+ (D) 1 min	Uptake error (SD) - K+ (D) 10 min	Uptake error (SD) - K+ (D) 60 min	Uptake error (SD) - K+ (D) 480 min
1	2	26	SPTGHISKSKTPTPRDNDNNSISDE	2696.2638	3.81	8.05	8.03	8.01	8.00	8.04	0.03	0.04	0.03	0.06	0.05
2	27	37	RETWSGKVDFL	1336.6775	9.95	3.74	3.69	3.66	3.66	3.65	0.04	0.03	0.02	0.02	0.02
3	27	38	RETWSGKVDFLL	1449.7616	11.14	3.48	3.50	3.81	3.78	3.62	0.07	0.02	0.07	0.05	0.10
4	37	43	LLSVIGF	747.4531	12.91	0.72	1.51	2.66	3.03	3.03	0.01	0.02	0.03	0.05	0.02
5	44	53	AVDLANVWRF	1189.6244	12.58	1.28	1.38	1.79	2.29	3.03	0.04	0.04	0.04	0.04	0.03
6	47	56	LANVWRFPYL	1277.6921	13.38	0.16	0.19	0.31	0.50	1.41	0.02	0.03	0.02	0.05	0.07
7	48	56	ANVWRFPYL	1164.6080	12.92	0.14	0.13	0.14	0.25	0.88	0.04	0.03	0.03	0.05	0.04
8	50	56	VWRFPYL	979.5280	12.87	0.11	0.09	0.10	0.19	0.62	0.02	0.02	0.02	0.03	0.04
9	57	65	CYKNGGGAF	915.3909	6.10	0.38	0.54	0.95	1.36	1.80	0.05	0.03	0.04	0.05	0.07
10	59	72	KNGGGAFLVPYGIM	1422.7330	11.73	0.13	0.19	0.44	0.60	0.95	0.03	0.03	0.05	0.06	0.03
11	65	72	FLVPYGIM	938.4935	13.15	0.02	0.09	0.08	0.03	0.04	0.02	0.02	0.03	0.09	0.03
12	66	73	LVPYGIML	904.5092	12.86	0.03	0.04	0.04	0.15	0.14	0.02	0.02	0.02	0.01	0.03
13	73	90	LVVGGIPLFYMELALGQH	1956.0543	10.98	4.54	4.93	5.50	6.34	7.11	0.07	0.05	0.06	0.08	0.03
14	76	85	GGIPLFYMEL	1138.5732	9.88	2.88	3.19	3.53	4.30	5.17	0.03	0.03	0.06	0.07	0.04
15	111	119	YAVVLIAFY	1057.5848	8.06	0.07	0.06	0.06	0.22	0.70	0.02	0.02	0.02	0.03	0.05
16	111	132	YAVVLIAFYVDFYYNVIIAWSL	2641.3872	10.34	9.09	9.81	10.82	10.81	10.71	0.14	0.09	0.12	0.04	0.13
17	169	193	PVIGNYSDLYAMGNQSLLYNETYMN	2869.2939	5.31	8.90	8.82	8.86	8.83	8.84	0.07	0.02	0.05	0.04	0.02
18	174	199	YSDLYAMGNQSLLYNETYMNGSSLDT	2949.2685	5.46	8.12	8.00	7.97	7.97	7.99	0.03	0.05	0.02	0.02	0.07
19	197	215	LDTSAVGHVEGFQSAASEY	1966.8908	8.90	7.88	7.90	8.45	8.97	9.04	0.12	0.07	0.05	0.06	0.10
20	198	214	DTSAVGHVEGFQSAASE	1690.7434	7.30	6.32	6.62	6.71	6.83	6.85	0.06	0.05	0.05	0.05	0.05
21	198	215	DTSAVGHVEGFQSAASEY	1853.8068	8.54	6.86	6.87	7.30	7.79	7.79	0.11	0.05	0.05	0.07	0.05
22	202	214	VGHVEGFQSAASE	1316.5997	6.13	4.53	4.56	4.74	4.82	4.83	0.07	0.05	0.06	0.06	0.06
23	215	222	YFNRYILE	1116.5604	9.69	0.39	0.50	1.40	2.76	2.75	0.05	0.06	0.06	0.08	0.08
24	216	222	FNRYILE	953.4970	8.75	0.29	0.37	1.18	2.45	2.51	0.04	0.05	0.04	0.06	0.08
25	224	234	NRSEGIHDLGA	1167.5632	5.52	0.21	0.22	0.26	0.26	0.25	0.02	0.03	0.03	0.04	0.02
26	239	246	MALCLLIV	874.5020	6.67	0.37	0.31	0.25	0.29	0.67	0.05	0.03	0.04	0.05	0.10
27	246	269	VYLICYFSLWKGISTSGKVVWFTA	2767.4448	6.47	5.97	6.15	6.78	7.13	7.29	0.16	0.12	0.04	0.06	0.07
28	283	294	GLTLPGSFLGIQ	1201.6707	8.04	1.73	1.87	2.06	2.46	2.92	0.04	0.06	0.11	0.09	0.04
29	284	290	LTLPGSF	733.4010	10.70	0.78	0.86	0.90	1.03	1.15	0.02	0.04	0.05	0.03	0.07
30	296	303	YLTPNFSA	911.4389	9.42	1.94	2.00	2.06	2.08	2.07	0.04	0.05	0.03	0.02	0.06
31	302	309	SAIYKAEV	879.4702	6.32	1.56	2.23	3.77	3.87	3.81	0.02	0.04	0.02	0.05	0.03
32	302	318	SAIYKAEVWVDAATQVF	1896.9621	13.18	1.67	2.51	4.30	4.48	4.60	0.02	0.04	0.09	0.07	0.12
33	319	325	FSLGPGF	723.3592	11.63	0.22	0.26	0.27	0.30	0.58	0.03	0.03	0.03	0.03	0.04
34	320	328	SLGPGFGVL	845.4647	11.94	0.29	0.36	0.64	1.20	2.12	0.01	0.02	0.03	0.03	0.01
35	329	346	LAYASYNKYHNNVYKDAL	2146.0483	7.39	2.26	2.86	4.28	5.76	6.80	0.03	0.03	0.04	0.05	0.02
36	329	347	LAYASYNKYHNNVYKDALL	2259.1324	8.43	2.12	2.72	4.03	5.50	6.73	0.03	0.09	0.04	0.06	0.03
37	331	346	YASYNKYHNNVYKDAL	1961.9272	6.67	1.83	2.19	2.99	4.00	5.11	0.07	0.03	0.05	0.21	0.07
38	348	357	TSFINSATSF	1073.5029	10.52	0.21	0.29	0.74	1.12	2.12	0.04	0.03	0.04	0.03	0.11
39	351	357	INSATSF	738.3548	7.36	0.23	0.34	0.70	0.99	1.60	0.04	0.03	0.03	0.04	0.03
40	365	376	SVLGYMAHTLGV	1246.6380	13.04	0.18	0.33	0.95	1.69	2.81	0.04	0.03	0.05	0.07	0.03
41	368	380	GYMAHTLGVRIED	1460.7082	7.96	1.54	1.92	2.74	3.86	4.65	0.02	0.05	0.04	0.13	0.05
42	376	390	VRIEDVATEGPGLVF	1600.8460	11.15	3.50	3.79	4.63	5.85	6.50	0.05	0.03	0.02	0.09	0.02
43	377	390	RIEDVATEGPGLVF	1501.7776	10.79	2.47	2.70	3.32	4.85	5.41	0.05	0.10	0.05	0.05	0.07
44	381	390	VATEGPGLVF	988.5229	11.15	1.92	1.89	2.36	2.96	3.18	0.03	0.05	0.05	0.08	0.03
45	404	415	TFWALIFFMMLL	1531.7971	9.55	3.43	4.18	5.58	6.16	6.46	0.10	0.08	0.05	0.05	0.04
46	404	417	TFWALIFFMMLLTL	1745.9289	7.09	3.19	3.74	4.78	5.71	6.23	0.06	0.07	0.06	0.11	0.11
47	411	429	FMMLLTLGLDSSFGGSEAI	1987.9635	9.39	7.52	7.54	7.50	7.36	7.45	0.04	0.12	0.07	0.09	0.05
48	428	447	AIITALSDEFPKIKRNRELF	2360.3216	10.26	1.64	3.14	6.84	7.89	8.36	0.06	0.05	0.08	0.04	0.05
49	428	451	AIITALSDEFPKIKRNRELFVAGL	2700.5326	10.97	1.74	3.18	6.92	8.01	8.89	0.07	0.06	0.06	0.08	0.11
50	433	451	LSDEFPKIKRNRELFVAGL	2231.2426	9.31	1.41	2.04	3.28	3.69	3.83	0.04	0.05	0.02	0.10	0.06
51	469	480	YFFHLLDRYAAG	1471.7248	11.25	0.33	0.51	0.68	0.91	1.23	0.03	0.05	0.07	0.05	0.07
52	480	490	GYSILVAVFFE	1243.6489	5.28	4.96	4.90	4.89	4.94	4.87	0.05	0.04	0.04	0.01	0.05
53	494	503	VSWIYGTNRF	1241.6193	10.62	0.79	0.84	0.94	1.09	1.01	0.07	0.10	0.11	0.07	0.05
54	507	518	IRDMIGFPPGRY	1420.7285	9.06	0.74	0.69	0.72	0.84	0.86	0.09	0.03	0.03	0.08	0.08
55	540	546	IGYEPLT	791.4065	8.32	1.31	2.00	2.18	2.25	2.20	0.03	0.10	0.04	0.03	0.06
56	550	555	YVYPSW	813.3697	11.31	1.28	1.29	1.56	1.80	1.72	0.03	0.04	0.03	0.03	0.04
57	550	556	YVYPSWA	884.4068	10.95	1.38	1.41	1.75	2.25	2.40	0.00	0.01	0.02	0.05	0.04
58	556	562	ANALGWC	733.3217	11.01	0.19	0.21	0.31	0.59	1.09	0.04	0.03	0.04	0.03	0.04
59	561	567	WCIAGSS	722.3057	8.71	0.24	0.26	0.27	0.27	0.46	0.02	0.02	0.03	0.01	0.02
60	568	575	VVMIPAVA	798.4673	9.29	0.28	0.42	0.48	0.47	0.41	0.03	0.02	0.06	0.06	0.03
61	568	576	VVMIPAVAI	911.5514	11.40	0.26	0.38	0.44	0.45	0.37	0.03	0.03	0.04	0.03	0.03
62	569	576	VMIPAVAI	812.4830	10.81	0.28	0.41	0.49	0.52	0.58	0.02	0.01	0.03	0.06	0.03
63	569	577	VMIPAVAIF	959.5514	13.35	0.32	0.38	0.54	0.62	0.60	0.04	0.02	0.07	0.05	0.02
64	576	593	IFKLLSTPGSLRQRFTIL	2089.2412	11.00	3.47	3.97	5.47	6.46	7.06	0.08	0.07	0.14	0.10	0.09
65	577	589	FKLLSTPGSLRQR	1501.8729	7.23	3.07	3.11	3.85	4.89	5.18	0.07	0.07	0.02	0.02	0.05
66	577	590	FKLLSTPGSLRQRF	1648.9413	8.77	3.09	3.12	3.93	5.01	5.44	0.06	0.03	0.03	0.05	0.11
67	577	593	FKLLSTPGSLRQRFTIL	1976.1571	10.50	3.31	3.71	5.10	6.45	7.10	0.07	0.05	0.09	0.08	0.05
68	578	593	KLLSTPGSLRQRFTIL	1829.0887	9.48	3.33	3.83	5.25	6.50	7.11	0.04	0.02	0.03	0.07	0.09
69	580	593	LSTPGSLRQRFTIL	1587.9097	9.86	3.24	3.55	4.46	5.69	6.20	0.10	0.03	0.06	0.09	0.07
70	591	604	TILTTPWRDQQSMA	1646.8086	9.40	4.47	4.60	5.13	5.87	6.43	0.07	0.03	0.03	0.06	0.03
71	594	603	TTPWRDQQSM	1248.5557	6.89	3.47	3.55	3.73	3.69	3.69	0.07	0.06	0.06	0.08	0.07
72	604	613	AMVLNGVTTE	1033.5114	8.12	5.35	5.36	5.37	5.36	5.43	0.02	0.04	0.04	0.03	0.08
73	605	613	MVLNGVTTE	962.4743	7.81	4.29	4.23	4.23	4.22	4.26	0.06	0.06	0.06	0.06	0.08
74	605	615	MVLNGVTTEVT	1162.5904	8.60	4.66	4.64	4.66	4.62	4.66	0.03	0.02	0.03	0.03	0.02
75	605	616	MVLNGVTTEVTV	1261.6588	9.91	5.78	5.80	5.79	5.74	5.82	0.04	0.07	0.05	0.06	0.09
76	606	613	VLNGVTTE	831.4338	5.93	3.57	3.55	3.57	3.55	3.56	0.05	0.05	0.04	0.05	0.04
77	606	615	VLNGVTTEVT	1031.5499	7.08	4.99	5.02	5.06	5.08	5.05	0.04	0.04	0.03	0.03	0.02
78	608	615	NGVTTEVT	819.3974	5.21	4.07	4.12	4.15	4.14	4.13	0.04	0.04	0.01	0.02	0.03
79	614	624	VTVVRLTDTET	1232.6612	7.01	5.38	5.35	5.35	5.32	5.37	0.07	0.07	0.06	0.07	0.07
80	614	630	VTVVRLTDTETAKEPVD	1871.9840	6.69	7.94	7.88	7.88	7.90	7.87	0.09	0.08	0.09	0.10	0.09
81	615	624	TVVRLTDTET	1133.5928	6.17	4.84	4.79	4.85	4.84	4.87	0.04	0.04	0.05	0.05	0.04
82	616	630	VVRLTDTETAKEPVD	1671.8679	5.72	6.79	6.72	6.74	6.71	6.76	0.05	0.06	0.05	0.05	0.05
83	617	630	VRLTDTETAKEPVD	1572.7995	5.28	6.10	6.04	6.05	6.05	6.10	0.09	0.07	0.07	0.08	0.07
84	620	630	TDTETAKEPVD	1204.5459	3.77	4.53	4.55	4.44	4.44	4.53	0.04	0.07	0.05	0.05	0.05
85	624	630	TAKEPVD	758.3810	5.72	2.72	2.64	2.65	2.69	2.69	0.05	0.03	0.08	0.04	0.09

Supplementary Table 4. Time dependent [^{3}H]dopamine uptake

Intra-vesicular cation	Max uptake (normalized)	*k* (min^{-1})	R^2	n
K^+	100 ± 1	0.68 ± 0.05	0.93	4
Cs^+	20 ± 1	0.94 ± 0.2	0.55	3
$NMDG^+$	20 ± 1	0.99 ± 0.3	0.40	3

The maximal uptake and rate constants (*k*) estimated for time dependent uptake of [^{3}H]dopamine uptake fitted to a one phase association. Uptake is shown in Fig. 4b. Values are given as mean ± SEM.

Supplementary Table 5. Concentration dependent [^{3}H]dopamine uptake

Intra-vesicular $[K^+]$ (mM)	V_{max} (normalized)	K_m (µM)	R^2	n
200	99 ± 3	0.85 ± 0.08	0.97	4
150	53 ± 4	2.13 ± 0.38	0.95	3
100	35 ± 3	1.81 ± 0.40	0.92	3
100 no gradient	24 ± 4	2.20 ± 0.76	0.87	3
0	38 ± 9	4.46 ± 1.76	0.86	4

Maximum dopamine uptake and K_m estimates for concentration dependent [^{3}H]dopamine uptake over 3 min in the indicated intra-vesicular K^+ concentrations including the condition with equimolar K^+ across the membrane (100 no gradient). Uptake shown in Fig. 4c and d. Values are given as mean ± SEM.

Supplementary Table 6. V_{max} and estimated corresponding n of dDAT from PLs with 200 mM intravesicular K^+.

Experiment	V_{max} (pmol min^{-1})	n dDAT (pmol)	k_{cat} (min^{-1})
1	1.40 ± 0.07	1.53 ± 0.18	0.9
2	1.73 ± 0.06	1.29 ± 0.06	1.3
3	1.13 ± 0.04	1.58 ± 0.11	0.7
4	1.88 ± 0.07	1.44 ± 0.03	1.3
Average	1.53	1.46	1.1

V_{max} obtained with 200 mM intra-vesicular K^+ and the amount of substance (n) of dDAT estimated from B_{max} from [^{3}H]nisoxetine binding measured on the protein re-solubilized from proteoliposomes containing 200 mM intra-vesicular K^+. The estimate of n of dDAT presented is based on two assumptions: a counting efficiency of 50% for the 2450 MicroBeta2 microplate counter in SPA mode and that the [^{3}H]nisoxetine was not radiochemical degraded within the timeframe of use. Data are shown as mean ± SD from 4 experiments (1-4) conducted in triplicates. Data are provided as a Source Data file.

Supplementary Table 7. Data from violin plots and box plots of the rates of Na^+ and K^+ flux

Shown in	Fig. 5e	Fig. 5e and sup. Fig. 7	Fig. 5f and sup. Fig. 7	Fig. 5f and g	Fig. 5g	Fig. 5h	Fig. 5h
Indicator	Na^+	Na^+	Na^+	Na^+	Na^+	K^+	K^+
Intra-vesicular buffer	$NMDG^+$	$NMDG^+$	K^+	K^+	$NMDG^+$	K^+	K^+
Uptake buffer	+ DA	-DA	-DA	+DA	+DA	+DA	-DA
N of single liposomes	162	110	83	86	162	527	382
min	0.32848	0.0354	0.26196	0.6568	0.32848	4.7483	2.47995
max	97.2579	66.0510	62.6744	111.039	97.2579	234.148	148.166
med	7.9521	8.40318	8.3583	11.5472	7.9521	41.2122	33.767
q1	5.16279	5.24223	5.17187	7.03001	5.16279	28.1841	23.3564
q3	15.582	17.5887	16.9616	25.4925	15.582	57.906	46.4426

The N of single liposomes assayed for the violin plots and box plots in Fig. 5 and Supplementary Fig. 7. The min, max med, q1 and q3 from the rates shown in the box plots in Fig. 5 and Supplementary Fig. 7 in (I/s).

SUPPLEMENTARY REFERENCES

1 Nielsen, A. K. *et al.* Substrate-induced conformational dynamics of the dopamine transporter. *Nat Commun* **10**, 2714, doi:10.1038/s41467-019-10449-w (2019).
