# Supplementary material for: The dopamine transporter antiports potassium to increase the uptake of dopamine
Source: Nat Commun. 2022 May 4;13:2446. doi: 10.1038/s41467-022-30154-5 (PMC9068915; doi:10.1038/s41467-022-30154-5)
Supplement: Supplementary file 3 — Reporting Summary [file 41467_2022_30154_MOESM3_ESM.pdf]

nature portfolio

Corresponding author(s): Claus Juul Loland

Last updated by author(s): Mar 15, 2022

Reporting Summary

Nature Portfolio wishes to improve the reproducibility of the work that we publish. This form provides structure for consistency and transparency in reporting. For further information on Nature Portfolio policies, see our Editorial Policies and the Editorial Policy Checklist.

Statistics

For all statistical analyses, confirm that the following items are present in the figure legend, table legend, main text, or Methods section.

n/a	Confirmed	
☐	☒	The exact sample size (*n*) for each experimental group/condition, given as a discrete number and unit of measurement
☐	☒	A statement on whether measurements were taken from distinct samples or whether the same sample was measured repeatedly
☐	☒	The statistical test(s) used AND whether they are one- or two-sided<br>*Only common tests should be described solely by name; describe more complex techniques in the Methods section.*
☒	☐	A description of all covariates tested
☐	☒	A description of any assumptions or corrections, such as tests of normality and adjustment for multiple comparisons
☐	☒	A full description of the statistical parameters including central tendency (e.g. means) or other basic estimates (e.g. regression coefficient) AND variation (e.g. standard deviation) or associated estimates of uncertainty (e.g. confidence intervals)
☐	☒	For null hypothesis testing, the test statistic (e.g. *F*, *t*, *r*) with confidence intervals, effect sizes, degrees of freedom and *P* value noted<br>*Give P values as exact values whenever suitable.*
☒	☐	For Bayesian analysis, information on the choice of priors and Markov chain Monte Carlo settings
☒	☐	For hierarchical and complex designs, identification of the appropriate level for tests and full reporting of outcomes
☐	☒	Estimates of effect sizes (e.g. Cohen's *d*, Pearson's *r*), indicating how they were calculated

Our web collection on statistics for biologists contains articles on many of the points above.

Software and code

Policy information about availability of computer code

Data collection	MassLynx (v. 4.1, Waters), UNICORN (v. 5.31, Cytiva), CellSens (Olympus), Wallac MicroBeta Windows Workstation 1.0 SP2 (Perkin Elmer)
Data analysis	GraphPad Prism (v. 7.0 and 9.0), Microsoft Excel 2016 and 2010 (Microsoft), ProteinLynx Global Server (v. 3.0, Waters), DynamX (v. 3.0, Waters), HX-Express, PyMOL (The PyMOL Molecular Graphics System, Version 2.0 Schrödinger, LLC). TIRF, tracking and localization was treated using in-house developed python script: Code available on https://github.com/hatzakislab/Dopamine-Manuscript

For manuscripts utilizing custom algorithms or software that are central to the research but not yet described in published literature, software must be made available to editors and reviewers. We strongly encourage code deposition in a community repository (e.g. GitHub). See the Nature Portfolio guidelines for submitting code & software for further information.

Data

Policy information about availability of data

All manuscripts must include a data availability statement. This statement should provide the following information, where applicable:

- Accession codes, unique identifiers, or web links for publicly available datasets
- A description of any restrictions on data availability
- For clinical datasets or third party data, please ensure that the statement adheres to our policy

A reporting summary for this Article is available as a Supplementary Information file. The source data underlying the graphical representations in Fig. 1, 2, 3, 4 and 5, Suppl. Figs. 1, 3, 5, 6 and Supplementary Table 6 are provided as source data file. The source data underlying all HDX-MS results in Fig. 2, 3 and Supplementary Figure 2 are shown in Supplementary Table 3. HDX-MS data files have been deposited to the PRoteomics IDEntification (PRIDE) Database with the dataset identifier PXDXXXXX. Source data for all single vesicle results in Figure 5 and Supplementary Figure 4 can be found in the freely available UCPH erda database here:

March 2021

https://sid.erda.dk/sharelink/BWguZqT2kL

Field-specific reporting

Please select the one below that is the best fit for your research. If you are not sure, read the appropriate sections before making your selection.

☒ Life sciences ☐ Behavioural & social sciences ☐ Ecological, evolutionary & environmental sciences

For a reference copy of the document with all sections, see nature.com/documents/nr-reporting-summary-flat.pdf

Life sciences study design

All studies must disclose on these points even when the disclosure is negative.

Sample size	The sample size were chosen based on previous experience for the molecular pharmacology (Plenge et al. Nat. Commun., 2020) and HDX experiments (Nielsen et al. Nat. Commun., 2019). For the remaining experiments sample sizes were determined based on variability within experiments in the optimization phase before beginning data acquisition.
Data exclusions	In the binding and uptake experiments a data replicate was excluded if Prism 7.0 determined it as an outlier. Peptides were excluded from the MS data if they were insufficiently fragmented and/or if the mass error was above 10 ppm. From the TIRF recordings no data was excluded.
Replication	Reproducibility was ensured by using at least 2 different generations of cells, 2 different viral transfections, 2 different membrane preparation, 2 different protein purifications, 2 different liposome preparations, 2 different proteoliposome reconstitutions, 3-4 (denoted in the figure text as number of n) individual experiments conducted on separate days with freshly prepared dilution series. Each experiment was conducted in triplicates (unless otherwise stated). TIRF recordings were made from 6-12 individual surfaces containing proteoliposomes from at least 2 individual reconstitutions with protein from 2 individual purifications. All attempts of replication of experiments were successful.
Randomization	The pharmacological experiments herein are performed in random order. HDX samples were injected into the LC-MS system according to the measuring time point (i.e. each measuring time point was analyzed to completion before starting a new time point). Samples were injected in a random order within the individual time points. Otherwise, randomization is not applicable to the data.
Blinding	The investigators were not blinded during data collection as all data were measured and reported objectively. All obtained data was analyzed, and the difference between experimental groups was assessed with statistical tests.

Reporting for specific materials, systems and methods

We require information from authors about some types of materials, experimental systems and methods used in many studies. Here, indicate whether each material, system or method listed is relevant to your study. If you are not sure if a list item applies to your research, read the appropriate section before selecting a response.

Materials & experimental systems

n/a	Involved in the study	
☒	☐	Antibodies
☐	☒	Eukaryotic cell lines
☒	☐	Palaeontology and archaeology
☒	☐	Animals and other organisms
☒	☐	Human research participants
☒	☐	Clinical data
☒	☐	Dual use research of concern

Methods

n/a	Involved in the study	
☒	☐	ChIP-seq
☒	☐	Flow cytometry
☒	☐	MRI-based neuroimaging

Eukaryotic cell lines

Policy information about cell lines

Cell line source(s)	COS-7 cells from ThermoFisher Scientific were used for cell uptake assays. Sf9 cells from Expression Systems were used for the production of the recombinant baculovirus. The HEK293 suspension cell line Expi293F from ThermoFisher Scientific was used for expression of dDAT.
Authentication	None of the cell lines used were authenticated
Mycoplasma contamination	All cell lines were tested negative for mycoplasma contamination.
Commonly misidentified lines (See ICLAC register)	none.
